# FusionEncoder: identification of intrinsically disordered regions based on multi-feature fusion

**DOI:** 10.1093/bioinformatics/btaf362

**Published:** 2025-06-19

**Authors:** Sicen Liu, Shutao Chen, Tao Bai, Bin Liu

**Affiliations:** SMBU-MSU-BIT Joint Laboratory on Bioinformatics and Engineering Biology, Shenzhen MSU-BIT University, Shenzhen, Guangdong 518172, China; School of Computer Science and Technology, Beijing Institute of Technology, Beijing 100081, China; School of Computer Science and Technology, Beijing Institute of Technology, Beijing 100081, China; School of Mathematics and Computer Science, Yan'an University, Shaanxi 716000, China; SMBU-MSU-BIT Joint Laboratory on Bioinformatics and Engineering Biology, Shenzhen MSU-BIT University, Shenzhen, Guangdong 518172, China; School of Computer Science and Technology, Beijing Institute of Technology, Beijing 100081, China; Zhongguancun Academy, Beijing 100094, China

## Abstract

**Motivation:**

Intrinsic disorder regions (IDRs) play a significant role in diverse biological processes and are widely distributed in proteins. Thus, accurately predicting these regions is essential for analyzing protein structure and function. Amino acid feature extraction servers as a foundational process in the development of computational predictive models. Existing methods typically rely on traditional biological features (e.g. PSSM) or use pre-trained protein language models (PPLMs) to capture sequence semantic information, often resorting to straightforward feature concatenation. However, these approaches fail to capture the multi-semantic interactions between traditional biological features and PPLMs-based features.

**Results:**

In this study, we propose a method named FusionEncoder designed for the integration of traditional biological and PPLMs-based features of the protein. FusionEncoder is a fusion network built on a variant of long short-term memory (LSTM). We consider traditional biological features and PPLMs-based features to be two types of semantic inputs within a “multi-semantic” space. Traditional features are input into the cell state of the LSTM, while PPLMs-based features are fed into the input part. A fusion cell is then utilized to fuse these two types of features. This strategy leverages the capability of LSTM to encode long sequences, enhancing context-aware semantic learning of amino acid sequences. Finally, a transformer-based encoder layer is employed to predict the IDRs. Evaluation on four independent test datasets indicate that FusionEncoder obviously improves the accuracy of amino acid feature representation and achieves superior performance compared to the other existing methods.

**Availability and implementation:**

To facilitate accessibility for experimental researchers, a user-friendly and publicly available webserver for the FusionEncoder predictor has been deployed at http://bliulab.net/FusionEncoder/. FusionEncoder is expected to serve as a valuable tool for the accurate identification of IDRs.

## 1 Introduction

Regions of protein that lack stable 3D structures are referred to as intrinsically disorder regions (IDRs) (Dyson and Wright 2005, Deng *et al.* 2012). Despite lacking stable 3D structures, IDRs are functionally indispensable, participating in various biological processes, such as cell signaling (Iakoucheva *et al.* 2002), DNA regulation (Wright and Dyson, 2015), and post-translational modification (Zhou *et al.* 2018). Moreover, numerous IDRs have been implicated in the pathogenesis of various human diseases (Uversky *et al.* 2008), such as Alzheimer’s disease (Uversky *et al.* 2009), cancer (Iakoucheva *et al.* 2002), etc. Therefore, accurate identification of IDRs is essential for advancing our understanding of complex biological processes and holds significant promise for informing drug design. The identification of intrinsically disordered regions (IDRs) has been facilitated by experimental methods such as circular dichroism (Receveur-Bréchot *et al.* 2006), X-ray crystallography (Receveur-Bréchot *et al.* 2006), and nuclear magnetic resonance (Konrat 2014). However, traditional wet-lab techniques are not well-suited for high-throughput data due to their labor-intensive, costly, and time-consuming characteristics (Liu *et al.* 2024). Accordingly, a variety of computational models have been proposed for IDR identification, employing machine learning and deep learning methodologies to enhance predictive performance (Hanson *et al.* 2017, 2019, Liu *et al.* 2019, Tang *et al.* 2022, Meng *et al.* 2023, Wang *et al.* 2024a,b).

The extraction of informative amino acid features plays a pivotal role in constructing effective predictive models for identifying IDRs (Tang *et al.* 2023). Traditional biological features commonly utilized in this field include evolutionary profiles, energy-based contact potential data, physicochemical properties, and so on (Jiang *et al.* 2023). For instance, IDP-CRF (Liu *et al.* 2018) leverages PSSM, secondary structure, K-mer, and relative solvent accessibility as residue feature representations, which are input into conditional random fields (CRFs) to predict IDRs. AUCpreD (Wang *et al.* 2016) incorporates residue-related, evolutionary, and structural features, and utilizes a convolutional neural networks (CNNs) and CRF hybrid framework to improve the accuracy of IDR prediction. SPOT-Disorder (Hanson *et al.* 2017) employs a long short-term memory (LSTM) architecture, incorporating evolutionary profiles, predicted structural attributes, and physicochemical features to enhance its predictive capability. IDP-seq2seq (Tang *et al.* 2021) incorporates a semantic space learning framework, leveraging evolutionary information, predicted secondary structures, physicochemical attributes, and spatial structural data as input features. DeepIDP-2L (Tang *et al.* 2022) approaches the task by encoding long and short disordered proteins based on their respective characteristics, thereby creating a predictor tailored to disordered regions. Moreover, biological sequences exhibit inherent similarities to natural language (Gimona 2006). For instance, the distribution of word occurrences in language and the frequency of domain appearances in proteomes both adhere to Zipf's law (Strait and Dewey 1996). These parallels fundamentally support the applicability of natural language processing (NLP) techniques in the analysis of biological sequences. With the continuous development of pre-trained language models and their success in natural language processing tasks, these models have proven effective in capturing contextual information and learning underlying patterns from large-scale training datasets (Cheng *et al.* 2021, Wei *et al.* 2021). Protein sequences, composed of amino acids, can be viewed as a form of biological language. Therefore, leveraging pre-trained protein language models (PPLMs) for amino acid feature extraction can effectively capture contextual information within amino acid sequences and uncover latent patterns present in large-scale protein pre-training data. For instance, DeepDRP (Yang *et al.* 2023) integrates traditional evolutionary information, physicochemical properties, and PPLMs features to shape meaningful representations from amino acid sequences and predict disordered regions. DisoFlAG (Pang and Liu 2024) leverages protein semantic information based on PPLMs to predict disorder regions.

**Figure. 1.**
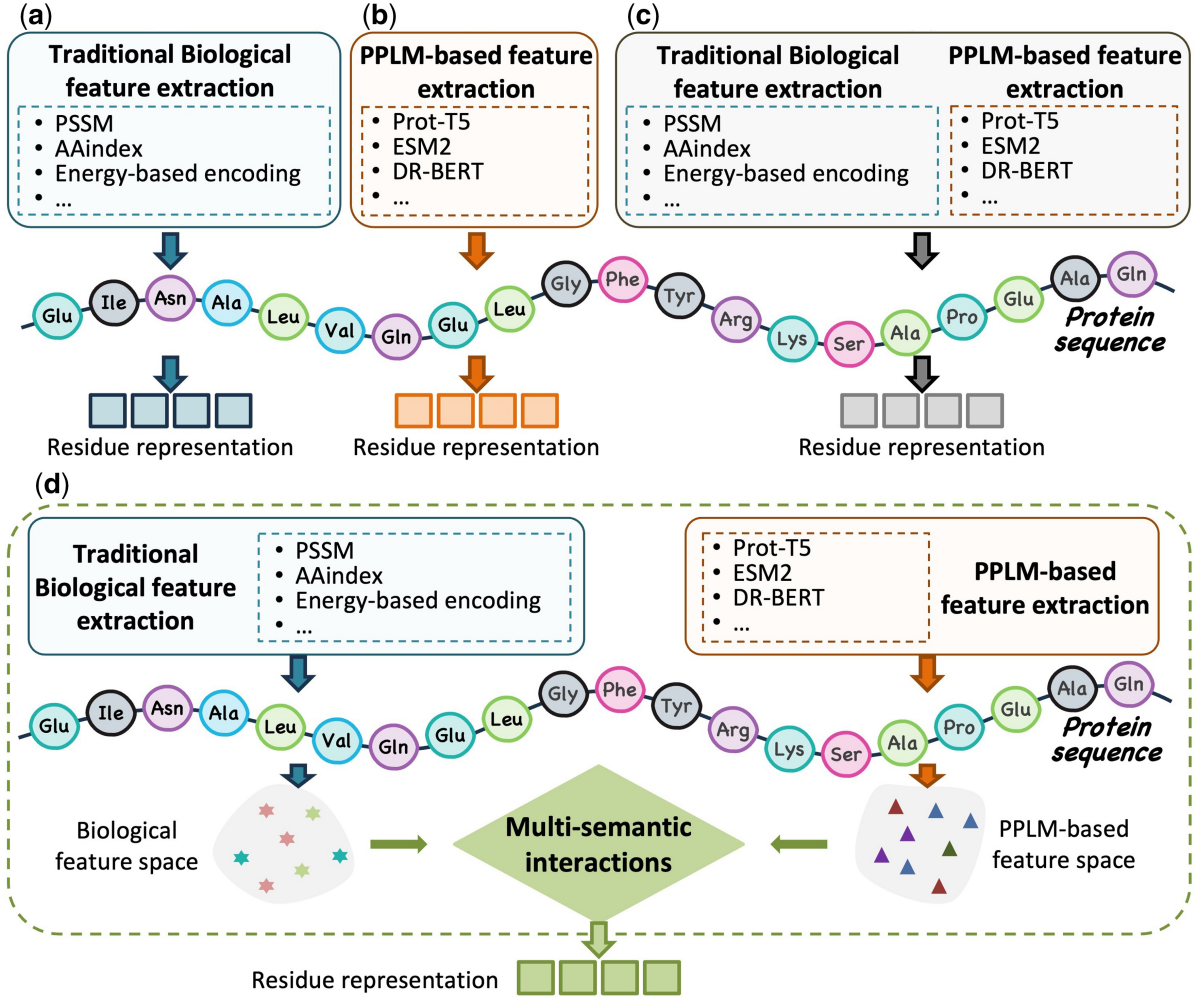
The differences between existing protein sequence feature extraction methods and the proposed FusionEncoder method. (a) Methods that rely on traditional biological property feature extraction. (b) Methods that rely on PPLM for protein sequence feature extraction. (c) Methods that directly combine traditional biological property feature and PPLMs-based protein sequence feature. (d) Our proposed FusionEncoder method, in which we perform multi-semantic interaction on the feature space based on traditional biological property extraction and the feature space based on PPLM extraction and then perform protein sequence feature extraction.

All these methods have advanced the development of predictive models for identifying IDRs. However, existing approaches still exhibit several limitations: (i) in feature selection, they often overlook the differences between traditional biological features and semantic features extracted by PPLMs, typically relying on either one or directly concatenating them for amino acid sequence representation. (ii) The effective integration of traditional biological and sequence semantic feature information across multi-semantic spaces, which would aid in more precise IDR identification, has not been sufficiently explored (see Fig. 1). Hence, integrating multi-semantic features and developing models that effectively capture semantic interactions across multi-semantic spaces for accurate IDR identification remains a challenging task.

In this study, we consider both traditional biological features of proteins and PPLMs-based features extracted using PPLMs as two distinct types of protein sequence inputs within a “multi-semantic” space. We employ an LSTM-based network framework to integrate these two “semantic” spaces, where the traditional biological features serve as guiding inputs to enhance the representations extracted by the PPLMs. Finally, an encoder layer is employed to model the amino acid sequences, capturing contextual dependencies to support accurate IDR prediction. Experimental evaluation across four independent test sets, along with a truncated test dataset, demonstrates that FusionEncoder outperforms existing computational methods, achieving both stable and superior performance.

## 2 Materials and methods

### 2.1 Training datasets

In this study, we employ a benchmark dataset constructed by Liu *et al.* (2021) for model evaluation. This dataset comprises 4845 protein sequences, including 4229 intrinsically disordered proteins and 616 ordered proteins. The ordered proteins were collected from the Protein Data Bank (PDB). Most existing IDP prediction models are developed and evaluated using datasets predominantly comprising fully disordered proteins, which may limit their generalizability to partially disordered cases. Proteins in nature consist of both disordered and ordered proteins. Therefore, we utilize a training dataset that includes both disordered and ordered proteins, which provides better generalization capability for the model. In detail, the disordered protein in the training dataset were collected from Zhang *et al.* (2012) with sequence similarity <25%. The ordered proteins satisfies following criteria: (i) each protein’s structure file contains a single chain, ensuring that no ordered regions are formed by the binding of IDRs with other proteins; (ii) the resolution of each protein is ≤2 and contains least 30 amino acids; (iii) the sequences similarity is below 25%; (iv) the atomic coordinates of each residue are documented in the PDB; and (v) nonstandard amino acids are excluded. The training dataset can be formatted as follows:

(1) $$S_{\text{Train}}=S_{\text{disordered}}\cup S_{\text{ordered}}.$$

To ensure a more reliable performance evaluation and improve the generalization capability of the computational model, we employed five-fold cross-validation to partition the training and validation sets.

### 2.2 Independent test datasets

For thorough performance assessment, two well-established datasets commonly used in IDR identification tasks were utilized: DISORDER723 (Sirota *et al.* 2010), MXD494 (Peng and Kurgan 2012). To further assess the generalization ability of various methods, FusionEncoder was also evaluated on the Disorder-Nox and Disorder-PDB test sets from round three of CAID (Necci *et al.* 2021). CAID3 Disorder-PDB annotations encompass intrinsically disordered residues detected by both circular dichroism and X-ray crystallography, while CAID3 Disorder-NOX annotations are restricted to residues identified exclusively through circular dichroism. Notably, residues absent from X-ray crystallography data are treated as unlabeled in the CAID3 Disorder-PDB annotation, whereas they are explicitly labeled as negative in the CAID3 Disorder-NOX annotation. Table 1 provides the statistical details of the four datasets.

**Table 1.** The statistical information of training and independent test datasets.

Dataset	Disordered residues	Ordered residues	Intrinsic disorder proteins	Ordered proteins
Training	103 252 (8.8%)	1 073 896 (91.2%)	4229 (87.3%)	616 (12.7%)
DISORDER723	13 526 (6.3%)	201 703 (93.7%)	723 (100%)	0 (0.0%)
MXD494	44 087 (22.4%)	152 414 (77.6%)	486 (98.4%)	8 (1.6%)
CAID3 Disorder-NOX	17 814 (24.5%)	55 010 (75.5%)	148 (100%)	0 (0.0%)
CAID3 Disorder-PDB	69 610 (59.5%)	47 443 (40.5%)	232 (100%)	0 (0.0%)

### 2.3 Residue feature representation

Residual feature extraction is a critical step in developing accurate and robust predictive models for IDR identification (Liu *et al.* 2014, Chen *et al.* 2017, Zhu *et al.* 2019, Zou *et al.* 2023). Two types of residue features were utilized in this study: (i) traditional biological features and (ii) semantic features extracted from PPLMs.

Among the traditional biological features, the position-specific scoring matrix (PSSM) (Altschul *et al.* 1997), AAindex (Kawashima *et al.* 2008), and energy-based encoding methods proposed by Dosztanyi *et al.* (2005) and Thomas and Dill (1996) are three of the most effective and commonly used approaches. The PSSM is employed to represent evolutionary conservation across protein sequences. AAindex reflects the physicochemical properties of amino acids. Additionally, we incorporated energy-based encoding features that represent the contact potentials between amino acids. The PSSM information was obtained by searching the NR90 (Holm and Sander 1998) database through three iterations of PSI-BLAST, with an *E*-value parameter set to 0.001, resulting in a PSSM-based feature dimension of 20. AAindex encompasses 566 unique physicochemical properties of amino acids. In line with the methodology from Yang *et al.* (2023), dimensionality reduction was performed using principal component analysis (PCA), yielding a set of 20 numerical values per amino acid. The energy-based encoding methods were utilized to represent the contact potentials between amino acids. Two paired energy groups were established, with each group containing 20 potential contact values for each residue. Thus, each amino acid corresponds to a 40-dimensional energy feature. Given a protein sequence P, the extracted traditional biological features can be represented as:

(2) $$P^{b}=R_{1}^{b},R_{2}^{b},\ldots ,R_{L}^{b},$$

where $R_{i}^{b}=[R_{i}^{\mathrm{PSSM}},\;R_{i}^{\mathrm{AAindex}},R_{i}^{\mathrm{energy}}]$, i∈{1,2,…,L} means the ith traditional biological residue represent vector and L is protein length. We concatenate the three types of traditional biological features into a single combined representation and [·,·] means the concatenate operation.

PPLMs-based semantic features effectively capture the contextual semantic information of protein sequences. In this study, we utilized four different PPLMs to extract various aspects of semantic information from protein sequences, including Prot-T5-XL-Uniref50 (Elnaggar *et al.* 2022), ESM-2 (Lin *et al.* 2023), DR-BERT (Nambiar *et al.* 2024), and OntoProtein (Zhang *et al.* 2022). Among these, Prot-T5, based on Google’s T5 framework, reformulates protein sequence modeling as a text-to-text generation task, facilitating unified and flexible representation learning. Its core concept treats amino acid sequences as a form of “language,” capturing evolutionary and functional patterns within the sequences through self-supervised learning, resulting in a semantic feature dimensionality of 1024 for each amino acid. ESM-2 is a transformer-based protein language model that focuses on learning evolutionary, structural, and functional information from amino acid sequences, with each amino acid yielding a feature dimensionality of 1280. DR-BERT is a lightweight protein language model tailored for IDR prediction, producing 768-dimensional semantic features for each amino acid. OntoProtein aims to enhance the model’s understanding of protein functions and evolution by explicitly incorporating domain knowledge, such as gene ontology (GO) and protein function annotations, with the extracted amino acid semantic feature dimensionality set at 30. Given a protein sequence P, the extracted PPLMs-based features can be represented as:

(3) $$P^{p}=R_{1}^{p},R_{2}^{p},\ldots ,R_{L}^{p},$$

where $R_{i}^{p}=[R_{i}^{\text{Prot}–T5},\;R_{i}^{\text{ESM}2},\;R_{i}^{\text{DR–BERT}},R_{i}^{\text{ontoProtein}}]$ is the ith PPLMs-based semantic residue feature vector (Zou *et al.* 2019), we concatenate the four types of PPLM-based features into a single combined representation and [·,·] means the concatenate operation.

### 2.4 Framework of FusionEncoder

**Figure 2.**
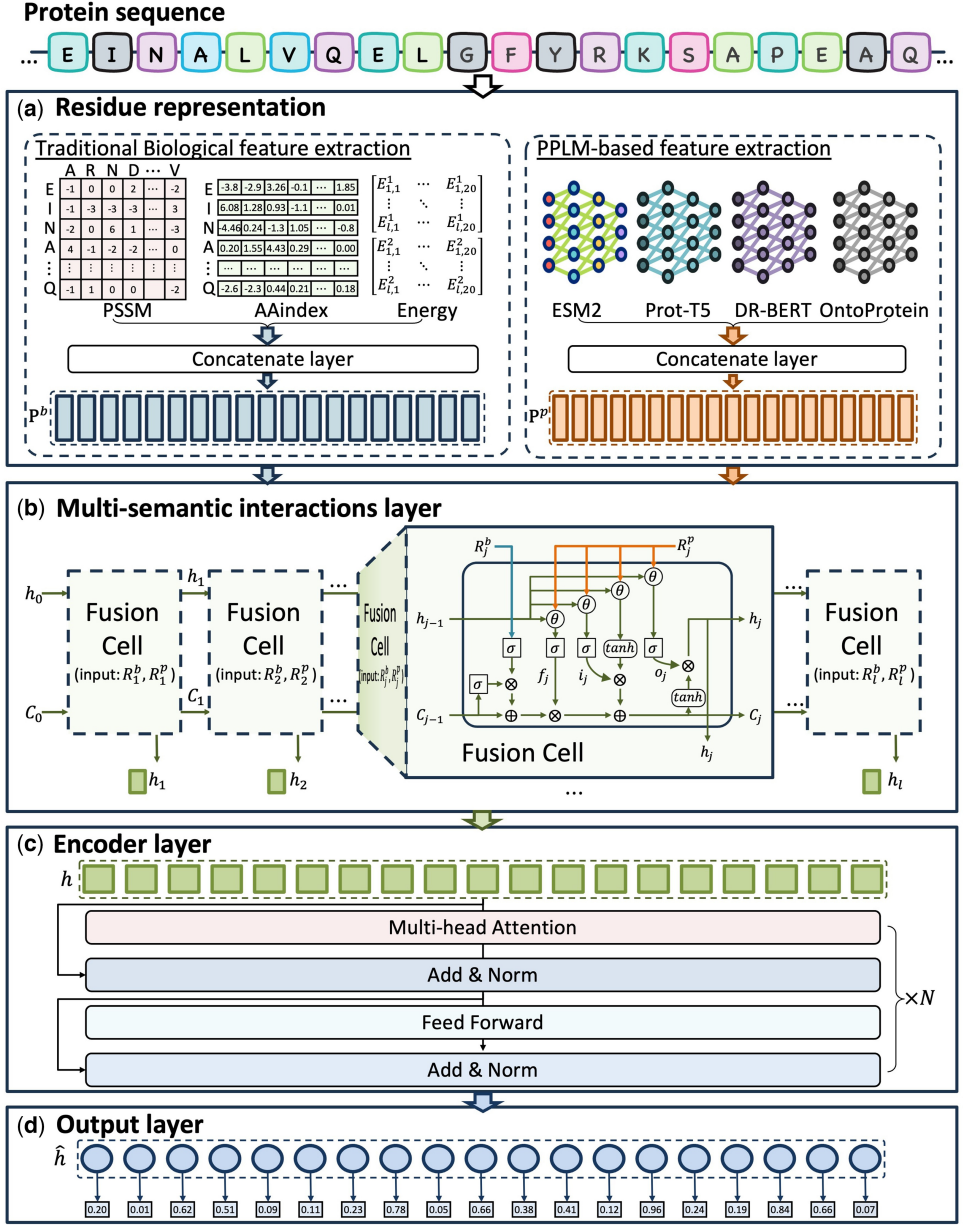
The framework of FusionEncoder. (a) The residue feature extraction process. This stage involves traditional biological feature extraction (PSSM, AAindex, and energy-based methods) and PPLMs-based feature extraction (ESM, ProtT5, DR-BERT, and OntoProtein methods). (b) Multi-semantic interaction layer. In this layer, traditional biological features and PPLMs-based features are fused using the FusionCell module, while long short-term memory (LSTM) facilitates information transfer between residues. (c) Encoder layer. A transformer-based encoder module is employed to encode protein sequences. (d) The output layer is utilized to perform predictions for IDRs.

In this work, FusionEncoder consists of three main deep neural network components, as illustrated in Fig. 2. First, the multi-semantic fusion layer integrates traditional biological features and PPLMs-based features for each residue using the proposed fusion cell module, while leveraging the characteristics of LSTM (Graves and Graves, 2012) to facilitate information transmission between residues. Second, the encoder layer employs a transformer-based encoding module to process the residue representations after multi-semantic feature fusion. Finally, the output layer is used to achieve accurate prediction of intrinsically disordered proteins.

#### 2.4.1 Multi-semantic interaction layer

To effectively integrate and interact between traditional biological and PPLMs-based features, we propose a fusion cell module that facilitates multi-semantic interaction. The fusion cell builds upon the gated memory cell of LSTM (Graves and Graves, 2012) , leveraging a gating mechanism to fuse traditional biological features with PPLMs-based features. Specifically, traditional biological features are incorporated into the memory cell as input from one semantic space, while PPLMs-based features serve as input from another semantic space, influencing the forget gate, input gate, and output gate. Ultimately, these elements work together to update the feature representation of the current residue, achieving multi-semantic interaction. The fusion of traditional biological features can be calculated by:

(4) $$C_{j-1}^{b}=\sigma (W_{c}C_{j-1}+b_{c})⊙\sigma (W_{r}R_{j}^{b}+b_{r}),$$

where $C_{j-1}$ denotes the memory cell of j−1th residue and $R_{j}^{b}$ represents the traditional biological feature of jth residue, ⊙ denotes Hadamard product, and $W_{c}$, $W_{r}$, $b_{c}$, $b_{r}$ denote the weight and bias.

The PPLMs-based features are fused together with the forget, input, and output gates, and together with the hidden state, they are used to update the candidate memory cell. The calculations for the gate and memory cell are as follows (Graves and Graves, 2012) :

(5) $$g_{j}=\sigma [R_{j}^{p},h_{j-1}]+b_{g},$$

 

(6) $${Ĉ}_{j}=\tanh\left(W_{ĉ}[R_{j}^{p},h_{j-1}]+b_{ĉ}\right),$$

where $g_{j}$ denotes three gates (forget, input, and output gates), $R_{j}^{p}$ denotes the PPLMs-based feature of jth residue, and $W_{g}$, $b_{g}$ are weight and bias. Finally, the memory cell and hidden state updates for the new jth residue follow the standard LSTM method. The final representation of the protein sequence is $h=[h_{j},\;h_{j},\;\ldots ,h_{j},\ldots ,\;h_{j}]$.

#### 2.4.2 Encoder layer

The encoder primarily consists of multi-head attention (MHA) mechanism and feed-forward layers (FFN). The protein sequence representation h, obtained after interaction in the multi-semantic layer, serves as the input to the encoder. The sublayer of encoder layer follow the transformer framework and calculated by （Vaswani *et al.* 2017):

(7) $$\text{MHA}(Q,K,V)=\text{Concate}({\mathrm{head}}_{1},{\mathrm{head}}_{2},\ldots ,{\mathrm{head}}_{k})W_{m},$$

 

(8) $$\text{FNN}(x)=\text{max}\left(0,xW_{1}+b_{1}\right)W_{2}+b_{2},$$

where ${\mathrm{head}}_{i}=\text{Attention}(QW_{i}^{Q},KW_{i}^{K},VW_{i}^{V})$ represents the computation of a single head attention, and i∈{1, 2, …, k}, Q, K, V represent query, key, and value of attention mechanism. The attention formulation is a variant of dot-product attention function (Bahdanau *et al.* 2015) and is calculated as follows:

(9) $$\text{Attention}(Q,K,V)=\mathrm{softmax}(\frac{{QK}^{T}}{\sqrt{d_{k}}})V,$$

where $d_{k}$ represents the feature dimension of the key vector. We used N sub-layers to encode the protein sequence and the final hidden state ĥ representation, obtained after encoding through the encoder layer, serves as the final representation of the protein sequence.

#### 2.4.3 Output layer

A fully connected layer followed by a softmax activation function is employed as the final classification module to generate residue-level predictions The classification threshold we used is 0.5, where residues with a predicted score greater than or equal to 0.5 are classified as disordered, and the remaining ones are classified as ordered. Detailed parameters of FusionEncoder are provided in Tables S1 and S2, available as supplementary data at *Bioinformatics* online.

### 2.5 Performance evaluation and metric

To assess the performance of various methods across different independent test datasets, we followed previous works (Liu *et al.* 2018,Tang *et al.* 2021, Zulfiqar *et al.* 2023, Guo *et al.* 2024, Huang *et al.* 2024, Zhu *et al.* 2024) and adopted five performance measures on the DISORDER723 and MXD494 independent test datasets, including Sn, Sp, BACC, MCC, and the area under the ROC curve (AUC)

(10) {Sn=TPTP+FNSp=TNTN+FPBACC=12(TPTP+FN+TNTN+FP)MCC=(TP×TN)-(FP×FN)(TP+FP)(TP+FN)(TN+FP)(TN+FN),

where TP, FP, TN, and FN denote the numbers of true positives, false positives, true negatives, and false negatives, respectively.

For the CAID3 disorder test datasets, we followed the official website (https://caid.idpcentral.org/challenge/results) and used the AUC, $F_{\max}$, and APS as evaluation metrics. *F*_max_ corresponds to the peak value on the precision–recall curve, AUC quantifies the area under the receiver operating characteristic (ROC) curve, and the average precision score (APS) is defined as the arithmetic mean of precision values along the precision–recall curve.

## 3 Results and discussion

### 3.1 FusionEncoder demonstrates stable performance across four independent test datasets

To compare the performance of FusionEncoder with other related methods, we conducted experimental validation on four independent test datasets: DISORDER723, MXD494, CAID3-NOX, and CAID3-PDB. The corresponding results are listed in Tables 2–5. The experimental results on DISORDER723 and MXD494 are obtained from Tang *et al.* (2023). By analyzing these tables, we gained the following observations: (i) FusionEncoder achieved the best performance on DISORDER723 and MXD494 test datasets in of BACC, MCC, and AUC (Tables 2 and 3). Specifically, on the DISORDER723 dataset, FusionEncoder outperformed the second-ranked method by 1.5% in BACC and 1.8% in AUC. On the MXD494 dataset, FusionEncoder outperformed IDP-Seq2seq in MCC by 1.7%. To further demonstrate the experimental performance of FusionEncoder, we conducted additional validation on the CAID3-NOX and CAID3-PDB datasets, with the corresponding results presented in Tables 4 and 5. Although our method did not achieve the best results in the disorder prediction task on CAID3, it ranked second in AUC on the CAID3-NOX test set, trailing the top-performing method by only 0.5%. Similarly, on the CAID3-PDB dataset, FusionEncoder ranked second in AUC, with a marginal 0.5% difference from the best-performing method. Additionally, FusionEncoder also ranked second in APS and Fmax on the CAID3-PDB test set, with only minor differences from the top-ranked method. These results demonstrate that FusionEncoder consistently delivers stable performance across different independent test datasets. (ii) The use of PPLMs-based semantic features can effectively improve the performance of disorder region prediction compared to methods that only use traditional biological features. On the DISORDER723 and MXD494 test datasets, FusionEncoder demonstrates more stable performance and better intrinsic disorder region prediction capabilities than methods that rely solely on traditional biological features, such as IDPFusion and DeepIDP-2L. These results further validate that feature selection is a key step in recognizing IDRs.

**Table 2.** Evaluation of various method on DISORDER723 independent test dataset.

Predictor^a^	Sn	Sp	BACC	MCC	AUC	Rank		
						AUC	BACC	MCC
FusionEncoder**^b^**	0.695	0.955	0.825	0.564	0.932	1	1	1
IDP-Fusion (Tang *et al.* 2023)	0.625	0.962	0.793	0.539	0.917	2	3	3
DeepIDP-2L (Tang *et al.* 2022)	0.615	0.962	0.789	0.529	0.914	3	4	6
AUCpredD (Wang *et al.* 2016)	0.580	0.974	0.777	0.564	0.914	3	6	1
IDP-Seq2Seq (Tang *et al.* 2021)	0.618	0.955	0.787	0.511	0.906	5	5	8
DISOPRED3 (Jones and Cozzetto 2015)	0.452	0.986	0.719	0.536	0.899	6	9	4
SPOT-Disorder (Hanson *et al.* 2017)	0.470	0.983	0.726	0.531	0.898	7	8	5
RFPR-IDP (Liu *et al.* 2021)	0.522	0.974	0.748	0.517	0.898	7	7	7
SPINE-D (Zhang *et al.* 2012)	0.779	0.840	0.810	0.376	0.891	9	2	10
IUPred-Short (Dosztányi *et al.* 2005)	0.495	0.943	0.719	0.382	0.810	9	10	9
IUPred-Long (Dosztányi *et al.* 2005)	0.298	0.949	0.623	0.247	0.721	11	11	11

a The result of IDF-Fusion was obtain from Tang *et al.* (2023), the result of DeepIDP-2L was obtain from Tang *et al.* (2022), and the result of IDP-Seq2Seq was obtain from Tang *et al.* (2021). Performance results of SPOT-Disorder, SPINE-D, AUCpreD, DISOPRED3, and RFPR-IDP were retrieved from their corresponding official websites or standalone implementations. The results for all other methods were obtained from Zhang *et al.* (2012).

^b^ The FusionEncoder implement details are list in Supplementary Tables S1 and S2.

**Table 3.** Evaluation of various method on MXD494 independent test dataset.

Predictor^a^	Sn	Sp	BACC	MCC	AUC	Rank		
						AUC	BACC	MCC
FusionEncoder**^b^**	0.742	0.806	0.774	0.492	0.842	1	1	1
IDP-Fusion (Tang *et al.* 2023)	0.712	0.808	0.760	0.470	0.834	2	3	3
DeepIDP-2L (Tang *et al.* 2022)	0.737	0.776	0.757	0.452	0.825	3	4	6
IDP-Seq2Seq (Tang *et al.* 2021)	0.743	0.791	0.767	0.475	0.825	3	2	2
MFDp (Mizianty *et al.* 2010)	0.746	0.768	0.757	0.451	0.821	5	4	7
MD (Schlessinger *et al.* 2009)	0.673	0.813	0.743	0.444	0.821	5	8	8
RFPR-IDP (Liu *et al.* 2021)	0.749	0.758	0.754	0.442	0.821	5	6	9
SPOT-Disorder (Hanson *et al.* 2017)	0.626	0.851	0.739	0.457	0.813	8	10	5
SPINE-D (Zhang *et al.* 2012)	0.787	0.698	0.742	0.411	0.803	9	9	11
AUCpredD (Wang *et al.* 2016)	0.521	0.881	0.701	0.411	0.800	10	16	11
DISOPRED3 (Jones and Cozzetto 2015)	0.622	0.820	0.721	0.410	0.800	10	13	13
IDP-FSP (Liu *et al.* 2019)	0.670	0.831	0.751	0.465	0.794	12	7	4
PONDER-FIT (Xue *et al.* 2010)	0.631	0.821	0.726	0.419	0.790	13	11	10
IUPred-long (Dosztányi *et al.* 2005)	0.522	0.866	0.694	0.389	0.781	14	14	15
DISOPRED2 (Ward *et al.* 2004)	0.647	0.800	0.724	0.406	0.781	14	12	14
IUPred-Short (Dosztányi *et al.* 2005)	0.522	0.866	0.694	0.389	0.781	14	17	16
DISpro (Cheng *et al.* 2005)	0.303	0.940	0.622	0.318	0.775	17	20	19
RONN (Yang *et al.* 2005)	0.664	0.754	0.709	0.368	0.764	18	15	17
Ucon (Schlessinger *et al.* 2007a,b)	0.554	0.787	0.671	0.313	0.741	19	19	20
NORSnet (Schlessinger *et al.* 2007a,b)	0.532	0.829	0.681	0.347	0.738	20	18	18
PROFbval (Schlessinger *et al.*, 2006)	0.835	0.387	0.611	0.196	0.697	21	21	21

a The result of IDF-Fusion was obtain from Tang *et al.* (2023), the result of DeepIDP-2L was obtain from Tang *et al.* (2022), and the result of IDP-Seq2Seq was obtain from Tang *et al.* (2021). Performance results of SPOT-Disorder, SPINE-D, AUCpreD, DISOPRED3, and RFPR-IDP were retrieved from their corresponding official websites or standalone implementations. The results for all other methods were obtained from Zhang *et al.* (2012).

^b^ The FusionEncoder implement details are list in Supplementary Tables S1 and S2.

**Table 4.** Performance of different methods on CAID3 Disorder-NOX test dataset.^a^

Predictor	AUC	APS	$F_{\max}$	${\mathrm{Rank}}_{\mathrm{AUC}}$
FusionEncoder	0.863	0.661	0.653	2
ESMDisPred-2PDB	0.868	0.738	0.723	1
ESMDisPred-2	0.859	0.732	0.711	3
rawMSA-disorder	0.858	0.640	0.640	4
DisoFLAG-IDR	0.857	0.694	0.633	5
ESMDisPred-1	0.857	0.734	0.701	6
flDpnn3a	0.851	0.687	0.642	7
DisorderUnetLM	0.850	0.689	0.615	8
rawMSA	0.847	0.655	0.614	9
udonPred-combined	0.841	0.617	0.627	10
flDPnn3b	0.836	0.644	0.615	11

a For a fair comparison, we directly obtained the experimental results of different predictors from the official CAID3 website (https://caid.idpcentral.org/challenge/results) and presented the performance of the top 10 computational methods based on AUC on the CAID3 Disorder-NOX test dataset.

**Table 5.** Performance of various methods on CAID3 Disorder-PDB test dataset.^a^

Predictor	AUC	APS	$F_{\max}$	${\mathrm{Rank}}_{\mathrm{AUC}}$
FusionEncoder	0.951	0.925	0.864	2
PUNCH2	0.956	0.927	0.865	1
PUNCH2-Light	0.950	0.925	0.862	3
AlphaFold3-rsa	0.949	0.906	0.853	4
AlphaFold-rsa	0.947	0.905	0.851	5
SPOT-Disorder2	0.945	0.910	0.831	6
LMDisorder	0.940	0.612	0.824	7
PredIDR2-Seq-Art	0.939	0.809	0.802	8
AlphaFold-pLDDT	0.938	0.902	0.841	9
PredIDR2-Prof-Art	0.935	0.866	0.785	10
SETH-0	0.933	0.905	0.843	11

a For a fair comparison, we directly obtained the experimental results of different predictors from the official CAID3 website (https://caid.idpcentral.org/challenge/results) and presented the performance of the top 10 computational methods based on AUC on the CAID3 Disorder-PDB test dataset.

### 3.2 Integrating traditional biological features with PPLMs-based features can improve prediction performance

Traditional biological features and PPLMs-based features are two key approaches for representing residues. While PLMs hold great potential, there is no clear evidence that they consistently outperform traditional biological feature-based methods in IDR prediction, nor has the optimal embedding method for IDR prediction been established. FusionEncoder utilizes a multi-semantic interaction layer to integrate traditional biological features with PPLMs-based features. To better validate the performance advantages of the multi-semantic interaction layer, we compared the experimental results with the DeepDRP (Yang *et al.* 2023) method, which also employs both traditional biological features and PPLMs-based features. In DeepDRP, traditional biological features and PPLM features are directly concatenated, as shown in Fig. 1c. DeepDRP truncates all protein features in the test set to a maximum length of 500, applying a masking strategy for sequences shorter than this limit. This ensures that all protein sequences have a length of 500 for input. To ensure a fair comparison, we adopted the same data settings as DeepDRP. The test dataset for DeepDRP was generated by truncating protein sequences based on the DISORDER723 dataset. The experimental results are presented in Table 6, with results of DeepDRP obtained from the original paper. It is evident that under the same data settings and feature inputs, our proposed FusionEncoder method demonstrates significantly better performance in predicting IDRs. Additionally, on the CAID3-NOX test dataset, compared to other methods that use PPLMs-based features [such as DisoFAG-IDR (Pang and Liu 2024)], the proposed FusionEncoder method also achieved strong performance. We also proposed removing the fusion cell to further demonstrate the effectiveness of integrating traditional biological features and PPLMs-based features. The corresponding method is denoted as FE_w/o Fusioncell_. As seen in Table 6, directly concatenating traditional biological features and PPLMs-based features, similar to DeepDRP, results in a noticeable performance drop in IDR prediction when using the standard LSTM method for residue sequence representation. This further highlights the effectiveness of FusionEncoder in integrating traditional biological features and PPLMs-based features. On the CAID3-PDB test dataset, PUNCH2 (Meng and Pollastri 2025) utilizes a combination of One-Hot, MSA-based, and PLM-based embeddings. It is clear that combining traditional biological features with PPLMs-based methods can obviously enhance IDR prediction performance. Moreover, our proposed FusionEncoder method consistently achieves stable results on both the CAID3-NOX and CAID3-PDB test datasets.

**Table 6.** Performance of different methods on truncated test dataset.^a^

Predictor	Sn	Sp	*F*1	MCC	AUC
FusionEncoder	0.5913	0.9821	0.6376	0.6172	0.9334
DeepDRP	0.4772	0.9914	0.5952	0.5953	0.9234
FE_w/o Fusioncell_	0.4966	0.9890	0.5989	0.5917	0.9280

a For a fair comparison, this truncated dataset is derived from the one used in Yang *et al.* (2023) paper. FE_w/o Fusioncell_ means that we replaced the Fusion cell with a standard LSTM cell, using the concatenated traditional biological features and PPLMs-based features as inputs.

### 3.3 Different feature extraction methods have impact on prediction performance

**Figure 3.**
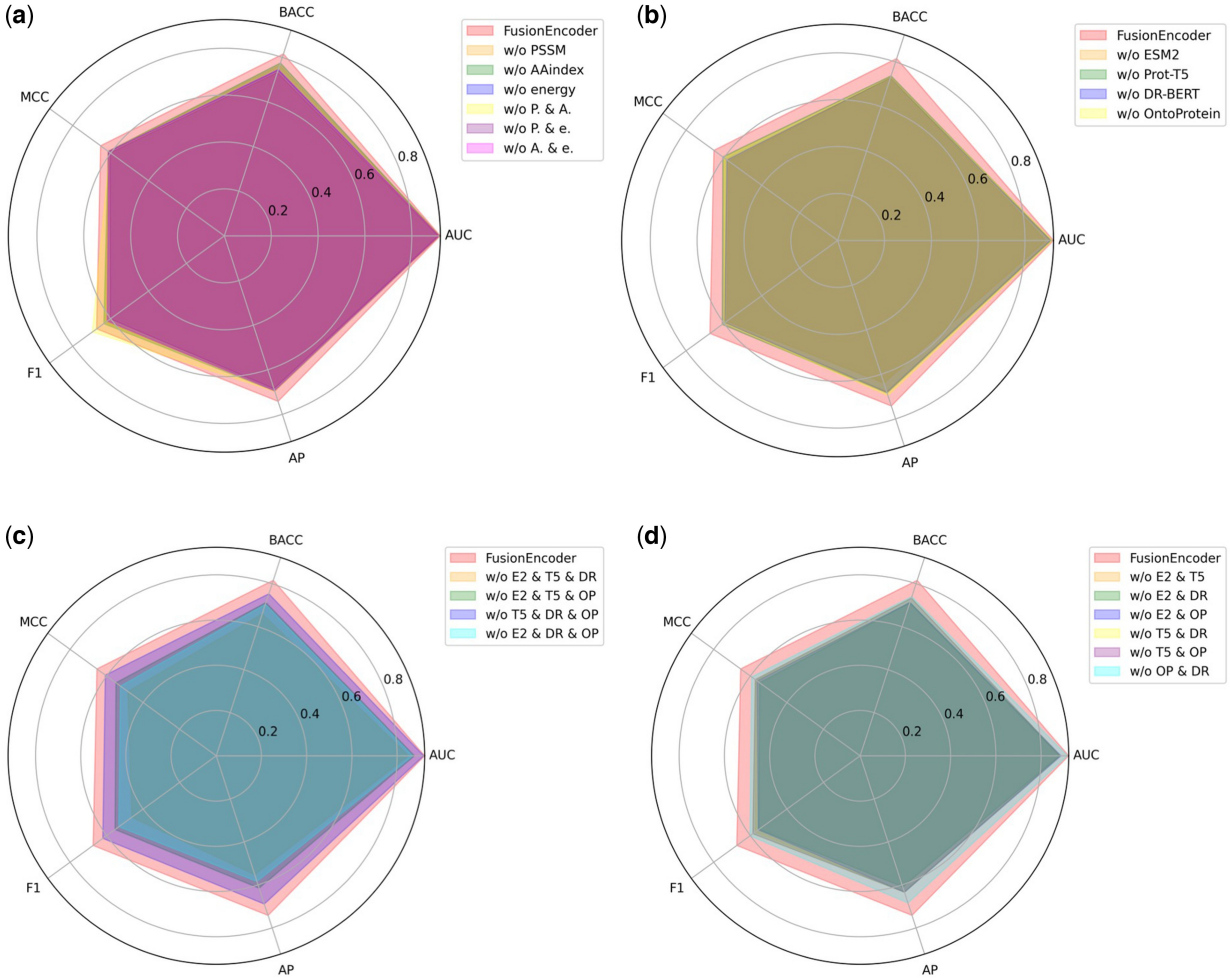
Ablation study of various feature combination on the validation dataset. To make the figure more compact, abbreviations were used to conserve space. P means PSSM, A means AAindex, e means energy, E2 means ESM2, T5 means Prot-T5, DR means DR-BERT, OP means OntoProtein, w/o means without.

FusionEncoder incorporates three different traditional biological features and four PPLMs-based features. To better understand the impact of different input features on model performance, we conducted ablation experiments on various feature combinations using the validation dataset. Five evaluation metrics were employed, with AP denoting average precision score. The results are presented in Fig. 3. In Fig. 3a, we show the results of ablation experiments on traditional biological features. A slight decrease in AUC was observed when individual traditional biological features were removed. Although the removal of different combinations of these features did not significantly affect AUC, notable declines were observed in other performance metrics. This suggests that, despite all being categorized as traditional biological features, different extraction methods within this category contribute differently to the prediction of IDRs. Figure 3b–d presents the ablation results for PPLMs-based features. As shown, there are substantial differences in IDR prediction performance across different PPLMs. Among them, the removal of ESM-2-derived features caused the model’s AUC to drop markedly from 0.922 to 0.889 (see Supplementary Table S4), indicating that the semantic features extracted using ESM2 have the most pronounced impact on model performance. The removal of other PPLMs-based features also led to varying degrees of performance degradation, further underscoring their individual contributions to the model’s predictive capacity. Taken together, the results in Fig. 3 suggest that while traditional biological features have a relatively modest impact on overall model performance, they are not negligible. In contrast, PPLMs-based features contribute more substantially to predictive accuracy. We hypothesize that this difference arises from the nature of PPLMs, which are pre-trained on large-scale datasets using unsupervised learning strategies to support downstream tasks. The primary goal of employing such pre-trained models is to capture as many latent features as possible, thereby enhancing the model’s representational power. To better illustrate the impact of different feature combinations, we quantitatively report model performance on the validation set using all five evaluation metrics. The results, shown in Tables S3 and S4, available as supplementary data at *Bioinformatics* online, indicate that although traditional biological features may contribute less to AUC improvement compared to PPLM-based features, they still positively influence overall model performance—particularly in metrics such as BACC, MCC, and F1.

### 3.4 Visualization of features at multi-semantic interaction layer

**Figure 4.**
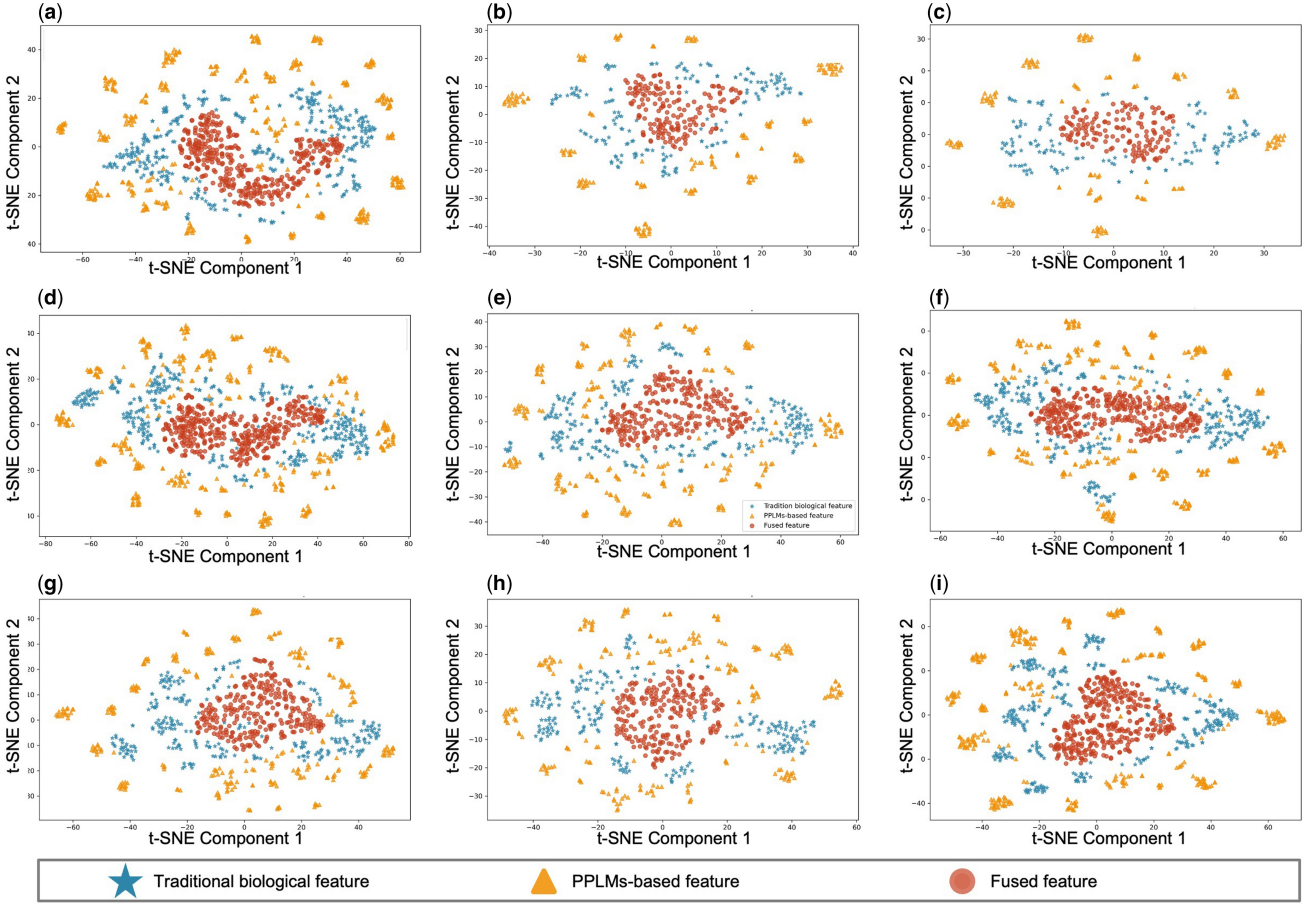
The t-SNE visualization of nine protein representations from DISORDER723 dataset.

FusionEncoder uses the fusion cell in the multi-semantic interaction layer to capture the interaction between traditional biological features and PPLMs-based features in residue sequences. To investigate how FusionCell facilitates semantic space interaction between conventional biological features and PPLMs-based semantic representations—thereby improving residue-level feature representation—we applied the t-SNE (Van Der Maaten and Geoffrey , 2008) dimensionality reduction technique on the DISORDER723 dataset. We randomly selected nine protein sequences [(Fig. 4a, PDB ID:1DB3), (Fig. 4b, PDB ID:1J79), (Fig. 4c, PDB ID:1JT9), (Fig. 4d, PDB ID:1JMX), (Fig. 4e, PDB ID:1BDF), (Fig. 4f, PDB ID:1EGD), (Fig. 4g, PDB ID:1FTK), (Fig. 4h, PDB ID:1GHE), (Fig. 4i, PDB ID:1I21)] for t-SNE visualization. As shown in Fig. 4, we observed that the distribution of both traditional biological features and PPLMs-based features became more compact after processing through the fusion cell. This indicates that the proposed fusion module effectively facilitates the integration of features originating from distinct semantic spaces, thereby enabling the generation of more informative and discriminative residue representations. Additionally, compared to PPLMs-based features, traditional biological features tend to exhibit a tighter, more clustered distribution, whereas PPLMs-based features are more widely dispersed, often occupying the outer regions of the 2D space. The representations based on traditional biological features and PPLMs-based features also exhibited substantial differences in their distribution within the 2D space. These observations further support the results shown in Fig. 1, demonstrating that our proposed multi-semantic interaction mechanism enables effective fusion and interaction between features originating from distinct semantic spaces—namely, traditional biological features and PPLMs-based features.

## 4 Conclusion

Amino acid feature extraction is a critical step in developing predictive models for IDRs. Traditional biological features and the emerging PPLMs-based features represent two widely used approaches for encoding amino acid sequences. Existing computational methods for IDR prediction typically rely on one of these representations or directly concatenate both, overlooking the potential semantic interactions between traditional biological features and PPLMs-based features. To address this limitation, we propose a novel method, FusionEncoder, aimed at enhancing IDR prediction performance. Specifically, we introduce a multi-semantic interaction layer to effectively integrate traditional biological features with PPLMs-based features, generating residue representations enriched with complementary semantic information. These fused features are then fed into an encoder layer to capture contextual semantics within protein sequences, followed by IDR prediction. Experimental results on four independent benchmark datasets demonstrate the effectiveness of the proposed FusionEncoder. The corresponding web server has been developed and is freely accessible at: http://bliulab.net/FusionEncoder/.

## Supplementary Material

btaf362_Supplementary_Data
