# Supplementary material for: FusionEncoder: identification of intrinsically disordered regions based on multi-feature fusion
Source: Bioinformatics. 2025 Jun 19;41(7):btaf362. doi: 10.1093/bioinformatics/btaf362 (PMC12231546; doi:10.1093/bioinformatics/btaf362)
Supplement: btaf362_Supplementary_Data [file btaf362_supplementary_data.docx]

Supplement file.

**Table S1**. The model detail parameters of FusionEncoder. The hidden size (HIDDEN_SIZE) varies across different datasets, with detailed hyperparameter settings provided in **Table S2**.

| Modules |  | Parameters |
| --- | --- | --- |
| Multi-semantic Interaction Layer | Input size | Traditions feature input size: 80  PPLMs-based feature input size:3102 |
|  | Fusion Cell | (W_ii): Linear(in_features=3102, out_features=HIDDEN_SIZE, bias=False)  (W_if): Linear(in_features=3102, out_features=HIDDEN_SIZE, bias=False)  (W_og): Linear(in_features=3102, out_features=HIDDEN_SIZE, bias=False)  (W_ig): Linear(in_features=3102, out_features=HIDDEN_SIZE, bias=False)  (W_aux): Linear(in_features=80, out_features=HIDDEN_SIZE, bias=False)  (U_ii): Linear(in_features=HIDDEN_SIZE, out_features=HIDDEN_SIZE, bias=False)  (U_if): Linear(in_features=HIDDEN_SIZE, out_features=HIDDEN_SIZE, bias=False)  (U_og): Linear(in_features=HIDDEN_SIZE, out_features=HIDDEN_SIZE, bias=False)  (U_ig): Linear(in_features=HIDDEN_SIZE, out_features=HIDDEN_SIZE, bias=False)  (U_aux): Linear(in_features=HIDDEN_SIZE, out_features=HIDDEN_SIZE, bias=False) |
| Encoder Layer |  | (layers): ModuleList( (0): EncoderLayer( (attention): MultiHeadAttention( (values): Linear(in_features=HIDDEN_SIZE, out_features=HIDDEN_SIZE, bias=False) (keys): Linear(in_features=HIDDEN_SIZE, out_features=HIDDEN_SIZE, bias=False) (queries): Linear(in_features=HIDDEN_SIZE, out_features=HIDDEN_SIZE, bias=False) (fc_out): Linear(in_features=HIDDEN_SIZE, out_features=HIDDEN_SIZE, bias=True) ) (norm1): LayerNorm((HIDDEN_SIZE,), eps=1e-05, elementwise_affine=True) (norm2): LayerNorm((HIDDEN_SIZE,), eps=1e-05, elementwise_affine=True) (feed_forward): Sequential( (0): Linear(in_features=HIDDEN_SIZE, out_features=512, bias=True) (1): ReLU() (2): Linear(in_features=512, out_features=HIDDEN_SIZE, bias=True) ) (dropout): Dropout(p=0.1, inplace=False) ) (1): EncoderLayer( (attention): MultiHeadAttention( (values): Linear(in_features=HIDDEN_SIZE, out_features=HIDDEN_SIZE, bias=False) (keys): Linear(in_features=HIDDEN_SIZE, out_features=HIDDEN_SIZE, bias=False) (queries): Linear(in_features=HIDDEN_SIZE, out_features=HIDDEN_SIZE, bias=False) (fc_out): Linear(in_features=HIDDEN_SIZE, out_features=HIDDEN_SIZE, bias=True) ) (norm1): LayerNorm((HIDDEN_SIZE,), eps=1e-05, elementwise_affine=True) (norm2): LayerNorm((HIDDEN_SIZE,), eps=1e-05, elementwise_affine=True) (feed_forward): Sequential( (0): Linear(in_features=HIDDEN_SIZE, out_features=512, bias=True) (1): ReLU() (2): Linear(in_features=512, out_features=HIDDEN_SIZE, bias=True) ) (dropout): Dropout(p=0.1, inplace=False) ) ) |
| Output Layer |  | (output_layer): Linear(in_features=HIDDEN_SIZE, out_features=1, bias=True) |
| Optimization |  | Optimizer: AdamW , loss: BCEWithLogitsLoss |

**Table S2**. The hyperparameters of FusionEncoder on different dataset.

| Datasets | Hyperparameter |
| --- | --- |
| DISORDER723 | Learning rate: 0.001, weight_decay:0.0001, hidden_size:128 |
| MXD494 | Learning rate: 0.001, weight_decay:0.0001, hidden_size:256 |
| CAID3 Disorder-NOX | Learning rate: 0.001, weight_decay:0.0001, hidden_size:128 |
| CAID3 Disorder-PDB | Learning rate: 0.001, weight_decay:0.0001, hidden_size:128 |
| truncated test datase | Learning rate: 0.001, weight_decay:0.0001, hidden_size:128, protein_seq_length: 500 |

**Table S3**. Performance of different combinations of traditional biological features on the validation dataset. The P. means PSSM, A. means AAindex, e. means energy, E2 means ESM2, T5 means Prot-T5, DR means DR-BERT, OP means OntoProtein. w/o means without.

| Models | AUC | BACC | MCC | F1 | AP |
| --- | --- | --- | --- | --- | --- |
| FusionEncode | 0.922 | 0.816 | 0.653 | 0.674 | 0.742 |
| w/o PSSM | 0.919 | 0.765 | 0.613 | 0.627 | 0.684 |
| w/o AAindex | 0.918 | 0.775 | 0.613 | 0.636 | 0.696 |
| w/o energy | 0.915 | 0.744 | 0.610 | 0.616 | 0.692 |
| w/o P. & A. | 0.916 | 0.735 | 0.606 | 0.606 | 0.697 |
| w/o P. & e. | 0.914 | 0.733 | 0.603 | 0.602 | 0.685 |
| w/o A. & e. | 0.917 | 0.746 | 0.612 | 0.619 | 0.695 |

**Table S4**. Performance of different combinations of PPLMs-based features on the validation dataset. The P. means PSSM, A. means AAindex, e. means energy, E2 means ESM2, T5 means Prot-T5, DR means DR-BERT, OP means OntoProtein. w/o means without.

| Models | AUC | BACC | MCC | F1 | AP |
| --- | --- | --- | --- | --- | --- |
| FusionEncode | 0.922 | 0.816 | 0.653 | 0.674 | 0.742 |
| w/o ESM2 | 0.889 | 0.731 | 0.575 | 0.585 | 0.639 |
| w/o Prot-T5 | 0.913 | 0.738 | 0.606 | 0.609 | 0.684 |
| w/o DR-BERT | 0.909 | 0.730 | 0.594 | 0.596 | 0.680 |
| w/o OntoProtein | 0.917 | 0.731 | 0.600 | 0.599 | 0.695 |
| w/o E2 & T5 | 0.886 | 0.710 | 0.568 | 0.573 | 0.633 |
| w/o E2 & DR | 0.882 | 0.712 | 0.549 | 0.554 | 0.609 |
| w/o E2 & OP | 0.888 | 0.713 | 0.563 | 0.563 | 0.634 |
| w/o T5 & DR | 0.884 | 0.733 | 0.573 | 0.586 | 0.634 |
| w/o T5 & OP | 0.916 | 0.716 | 0.61 | 0.624 | 0.695 |
| w/o DR & OP | 0.919 | 0.74 | 0.593 | 0.602 | 0.684 |
| w/o E2 & T5 & DR | 0.851 | 0.6594 | 0.483 | 0.462 | 0.552 |
| w/o E2 & T5 & OP | 0.877 | 0.712 | 0.549 | 0.554 | 0.615 |
| w/o T5 & DR & OP | 0.918 | 0.753 | 0.607 | 0.621 | 0.689 |
| w/o E2 & DR & OP | 0.870 | 0.704 | 0.524 | 0.533 | 0.584 |
